# Exploring the Link: Marijuana Use Patterns and Their Impact on Coronary Heart Disease Risk

**DOI:** 10.1002/clc.70223

**Published:** 2025-12-05

**Authors:** Tianwen Wei, Shitong Shen, Tiankai Shan, Tangjiang Wan, Yucheng Liang, Zhihao Lin, Yuxiao Sun, Yafei Li, Qi Zhang

**Affiliations:** ^1^ Department of Cardiology, Shanghai East Hospital, School of Medicine Tongji University Shanghai PR China; ^2^ Department of Cardiology The Affiliated Suzhou Hospital of Nanjing Medical University, Suzhou Municipal Hospital, Gusu School, Nanjing Medical University Suzhou Jiangsu China; ^3^ Department of Cardiology The First Affiliated Hospital of Soochow University Suzhou Jiangsu China

**Keywords:** cardiovascular health, coronary heart disease, marijuana use, public health, risk factors

## Abstract

**Background:**

As marijuana use increases globally, understanding its cardiovascular impact is crucial. This study investigates the relationship between marijuana use patterns and coronary heart disease (CHD) risk.

**Methods:**

Using 2023 Behavioral Risk Factor Surveillance System data, we conducted cross‐sectional analysis examining marijuana consumption methods (smoking, vaping, eating, dabbing) and CHD risk. Multivariable logistic regression assessed associations while controlling for demographic variables and established cardiovascular risk factors.

**Results:**

Significant associations emerged between various marijuana consumption methods and increased CHD risk. Individuals engaging in both smoking and vaping showed significantly higher CHD risk. Combined consumption methods, particularly smoking and eating, further compounded risk among those with pre‐existing cardiovascular risk factors. Traditional risk factors including hypertension, diabetes, and obesity remained critical CHD predictors.

**Conclusion:**

This study reveals complex relationships between marijuana use patterns and CHD risk, emphasizing the need for comprehensive public health strategies addressing cardiovascular implications of marijuana consumption. As usage rises, particularly among younger populations, healthcare providers and policymakers must educate individuals about potential cardiovascular risks associated with different consumption methods. Further research is needed to elucidate mechanisms and inform future guidelines for CHD risk reduction.

## 1 Introduction

Coronary heart disease (CHD) remains a leading cause of morbidity and mortality worldwide, representing a significant public health challenge that demands ongoing research and intervention strategies [1]. This condition, characterized by the narrowing or blockage of coronary arteries, can lead to serious complications such as heart attacks and heart failure. Understanding the multifactorial nature of CHD is crucial for developing effective prevention and treatment strategies [2]. While traditional risk factors such as hypertension, diabetes, smoking, obesity, and sedentary lifestyle have been extensively studied, emerging research has begun to explore the role of various lifestyle choices, including substance use, in influencing cardiovascular health [3, 4, 5].

Among recent trends in lifestyle choices, marijuana use has gained considerable attention due to its increasing prevalence and changing legal status in many regions [6]. Once associated predominantly with recreational use, marijuana is now recognized for its potential therapeutic applications in managing a wide range of conditions, including chronic pain, anxiety disorders, and seizures [7, 8]. However, the changing landscape of marijuana use poses new questions regarding its long‐term health impacts, particularly on cardiovascular health [9]. Preliminary studies suggest that marijuana consumption may have complex effects on heart health, depending on factors such as method of use, frequency, and individual health conditions [10, 11].

The relationship between marijuana use and cardiovascular health is complex and not yet fully understood. While some studies indicate that marijuana may pose certain cardiovascular risks [12], particularly concerning heart rate and blood pressure, others suggest that its anti‐inflammatory properties may have protective effects for specific cardiovascular conditions [13]. This paradox necessitates careful examination of how different patterns of marijuana use may interact with traditional risk factors to influence the likelihood of developing CHD [14].

As the prevalence of marijuana use continues to rise, particularly among younger populations, there is an urgent need for comprehensive research that addresses the potential cardiovascular implications of various consumption methods, such as smoking, vaping, and edibles [15, 16]. Understanding how these consumption patterns may interact with established risk factors and alter the risk of CHD can contribute valuable insights to the existing body of literature and inform public health approaches.

The Behavioral Risk Factor Surveillance System (BRFSS) provides a unique opportunity to investigate these relationships on a national scale. Conducted annually, BRFSS collects self‐reported data from thousands of adults across the United States, encompassing a broad range of health‐related behaviors, chronic conditions, and demographic information. Utilizing this extensive dataset allows researchers to examine the interplay between marijuana use, traditional cardiovascular risk factors, and the occurrence of CHD on a population level [17, 18, 19].

Moreover, individual susceptibility to CHD is influenced by a variety of factors, including genetic predispositions, socioeconomic status, access to healthcare, and differing responses to environmental influences. These intricacies highlight the necessity of adopting a holistic approach to studying cardiovascular health, where lifestyle behaviors, health disparities, and risk perceptions are interconnected.

To address this knowledge gap, the current study seeks to explore the relationship between different patterns of marijuana use and the risk of CHD, while controlling for traditional cardiovascular risk factors. By employing a rigorous methodology and utilizing data from the BRFSS, this study aims to contribute to the understanding of how marijuana consumption may influence cardiovascular health on a population level. The findings from this study aim to inform public health policies and interventions designed to mitigate heart disease risk, particularly in populations where marijuana use is prevalent.

In summary, the intersection of marijuana use and cardiovascular health presents an intriguing area of inquiry that warrants further exploration. Given the rising rates of marijuana consumption and its evolving legal status, it is crucial to understand the implications of this lifestyle choice on CHD risk. By analyzing the interactions between various marijuana consumption practices and traditional cardiovascular risk factors, the current study aims to provide valuable insights that can enhance our understanding of heart health and guide public health strategies in managing and preventing coronary heart disease. The findings will not only contribute to the discourse surrounding marijuana use but also strive to clarify its role within a broader context of cardiovascular risk, ultimately aiming to promote healthier lifestyles and reduce the burden of heart disease in the population.

## 2 Methodology

### 2.1 Data Source

This study utilizes data from the BRFSS 2023 database. The BRFSS is an ongoing, state‐based, phone survey system that collects data on health‐related risk behaviors, chronic health conditions, and use of preventive services among adults aged 18 years and older in the United States. The database provides a representative sample of U.S. adults and serves as a valuable resource for public health research.

### 2.2 Study Population

The sample for this study consisted of adult participants from the BRFSS 2023 survey. The total number of completed interviews was 433 323. The analysis focused on individuals who provided information regarding their marijuana use patterns and responses related to CHD risk factors. Inclusion criteria required participants to have completed relevant sections of the survey, ensuring the availability of necessary data.

### 2.3 Variables

Dependent Variable:

Coronary Heart Disease: The primary outcome variable, assessed through survey questions regarding the participant's medical history of heart disease.

Independent Variables:

Marijuana Use Patterns:

Smoking marijuana (binary: Yes/No)

Vaping marijuana (binary: Yes/No)

Eating marijuana (binary: Yes/No)

Dabbing marijuana (binary: Yes/No)

Covariates:

Demographic Variables:

Age (continuous variable)

Gender (binary: Male/Female)

Race (categorical variable with categories such as White, Black, Asian, Native American, and Hispanic)

State (categorical variable based on state FIPS codes)

Socioeconomic Status:

Education Level (categorical: Less than high school, High school graduate, Some college, College graduate)

Health‐Related Factors:

BMI (categorical variable with Underweight, Normal Weight, Overweight, Obesity)

Smoking Status (binary: Current smoker/Nonsmoker)

Alcohol Consumption (binary: Binge drinker/Non‐binge drinker)

Physical Activity (measured as days of moderate and vigorous activities)

Comorbid Conditions:

High blood pressure (binary: Yes/No)

Diabetes (binary: Yes/No)

Cholesterol medication status (binary: Yes/No)

History of stroke (binary: Yes/No)

### 2.4 Statistical Analysis

The statistical analysis for this study was designed to investigate the relationship between marijuana use patterns and the risk of CHD using data from the 2023 BRFSS. The analysis consisted of several key steps outlined in detail below:

### 2.5 Data Preprocessing

Before conducting the statistical analyses, the dataset underwent several preprocessing steps:

#### 2.5.1 Missing Data

The proportion of missing data for each variable was examined. Cases with missing values for critical variables (such as CHD status or marijuana use patterns) were excluded from the analysis. For variables with less than 5% missing data, single imputation methods, such as mean or median substitution, were applied when appropriate.

#### 2.5.2 Outlier Detection

Outliers in continuous variables, such as age and BMI, were identified using z‐scores (a threshold of ±3 was applied). Observations identified as outliers were reviewed to determine data entry errors or valid extreme values.

### 2.6 Descriptive Statistics

Descriptive statistics were calculated to summarize the characteristics of the study population:

Frequencies and percentages were computed for categorical variables (e.g., marijuana use patterns, gender, race/ethnicity).

Means and standard deviations (SDs) were reported for continuous variables (e.g., age, BMI).

The demographic and health‐related characteristics of participants were presented in a table to provide a clear overview of the sample's composition.

### 2.7 Multivariable Logistic Regression Analysis

To evaluate the association between marijuana use patterns and the risk of CHD, a multivariable logistic regression analysis was performed. This analysis aimed to estimate the odds ratios (ORs) for the likelihood of having CHD based on different marijuana use patterns, adjusting for potential confounding variables:

#### 2.7.1 Covariates Included

The model controlled for demographic factors (age, gender, race/ethnicity), socioeconomic status (education level, income), health‐related risk factors (BMI, smoking status, physical activity), and comorbid conditions (such as high blood pressure and diabetes).

#### 2.7.2 Interaction Terms

Interaction terms were created to examine the combined effects of different marijuana consumption methods (e.g., Smoking × Vaping, Smoking × Eating) on CHD risk. These terms were added to the model to assess whether the risk associated with one type of consumption was modified by another type.

#### 2.7.3 Model Fitting

The logistic regression model was fit using software such as R or SAS. The fit of the model was assessed through the Hosmer‐Lemeshow goodness‐of‐fit test to ensure that the model sufficiently describes the observed data.

### 2.8 Weighting for BRFSS Design

The analysis used sampling weights provided by BRFSS to ensure that estimates reflect the distribution of the U.S. adult population accurately. Weights were applied during the regression analysis to adjust for unequal probabilities of selection and to account for nonresponse bias.

Weights were incorporated as follows:

The statistical software provided the option to specify weights during model fitting, ensuring that point estimates and confidence intervals were based on the weighted data.

### 2.9 Statistical Significance and Interpretation

The significance levels were set at *p* < 0.05 for all analyses. The ORs along with 95% CIs were reported for each variable in the logistic regression model. An OR greater than 1 indicated increased odds of having CHD, while an OR less than 1 indicated decreased odds.

Results were interpreted in the context of public health implications, where significant findings were emphasized in the discussion, particularly focusing on the interactions between different marijuana use patterns and their combined effect on cardiovascular health.

Overall, this rigorous analytical approach provided a comprehensive understanding of how various patterns of marijuana use interact to influence the risk of coronary heart disease, thereby contributing valuable insights for public health policies and interventions aimed at mitigating cardiovascular risks associated with cannabis consumption.

### 2.10 Statistical Software

All analyses were performed using R statistical software version 4.3.0. Data preprocessing and manipulation were conducted using the dplyr and tidyr packages, while descriptive statistics were generated with base R functions. Multivariable logistic regression analysis was carried out using the glm function from base R to estimate the association between marijuana use patterns and the risk of CHD. Interaction effects were assessed by including interaction terms within the logistic regression model.

## 3 Results

### 3.1 Demographic Characteristics

The study investigates the correlation between marijuana use and CHD among a total sample size of 7672 participants, with a primary focus on various characteristics such as age, gender, race, education level, BMI category, existing health conditions, and lifestyle factors (Table 1).

**TABLE 1:** Demographic characteristics and their relationship with marijuana use and coronary heart disease in a sample of 7672 participants.

Characteristics	None (*N* = 7255)	Yes (*N* = 417)	Total (*N* = 7672)	*p*‐value	FDR
Smoke Marijuana				0.008	0.022
None	2106 (27.45%)	104 (1.36%)	2210 (28.81%)		
Yes	5149 (67.11%)	313 (4.08%)	5462 (71.19%)		
Eating Marijuana				3.60E‐40	6.20E‐39
None	3824 (49.84%)	80 (1.04%)	3904 (50.89%)		
Yes	3431 (44.72%)	337 (4.39%)	3768 (49.11%)		
Vaping Marijuana				2.60E‐93	5.20E‐92
None	5574 (72.65%)	132 (1.72%)	5706 (74.37%)		
Yes	1681 (21.91%)	285 (3.71%)	1966 (25.63%)		
Dabbing Marijuana				7.90E‐59	1.40E‐57
None	6827 (88.99%)	305 (3.98%)	7132 (92.96%)		
Yes	428 (5.58%)	112 (1.46%)	540 (7.04%)		
Age Group				9.20E‐36	1.40E‐34
18–24	415 (5.41%)	14 (0.18%)	429 (5.59%)		
24–29	473 (6.17%)	14 (0.18%)	487 (6.35%)		
30–34	634 (8.26%)	10 (0.13%)	644 (8.39%)		
35–39	734 (9.57%)	11 (0.14%)	745 (9.71%)		
40–44	763 (9.95%)	24 (0.31%)	787 (10.26%)		
45–49	589 (7.68%)	19 (0.25%)	608 (7.92%)		
50–54	651 (8.49%)	36 (0.47%)	687 (8.95%)		
55–59	650 (8.47%)	31 (0.40%)	681 (8.88%)		
60–64	794 (10.35%)	70 (0.91%)	864 (11.26%)		
65–69	762 (9.93%)	79 (1.03%)	841 (10.96%)		
70–74	513 (6.69%)	62 (0.81%)	575 (7.49%)		
75–79	199 (2.59%)	29 (0.38%)	228 (2.97%)		
80–84	56 (0.73%)	16 (0.21%)	72 (0.94%)		
85+	22 (0.29%)	2 (0.03%)	24 (0.31%)		
Gender				7.80E‐08	8.60E‐07
Female	3281 (42.77%)	132 (1.72%)	3413 (44.49%)		
Male	3974 (51.80%)	285 (3.71%)	4259 (55.51%)		
Race				0.03	0.11
American Indian	149 (1.94%)	16 (0.21%)	165 (2.15%)		
Asian	67 (0.87%)	0 (0.0e + 0%)	67 (0.87%)		
Black	479 (6.24%)	19 (0.25%)	498 (6.49%)		
Multiracial	226 (2.95%)	18 (0.23%)	244 (3.18%)		
Native Hawaiian	57 (0.74%)	4 (0.05%)	61 (0.80%)		
Other race	47 (0.61%)	3 (0.04%)	50 (0.65%)		
White	6230 (81.20%)	357 (4.65%)	6587 (85.86%)		
Education Level				0.02	0.09
College Graduate	3224 (42.02%)	154 (2.01%)	3378 (44.03%)		
High School Graduate	1677 (21.86%)	107 (1.39%)	1784 (23.25%)		
Less than High School	276 (3.60%)	22 (0.29%)	298 (3.88%)		
Some College	2078 (27.09%)	134 (1.75%)	2212 (28.83%)		
BMI Category				3.90E‐03	0.03
Normal Weight	2265 (29.52%)	100 (1.30%)	2365 (30.83%)		
Obesity	2288 (29.82%)	157 (2.05%)	2445 (31.87%)		
Overweight	2566 (33.45%)	148 (1.93%)	2714 (35.38%)		
Underweight	136 (1.77%)	12 (0.16%)	148 (1.93%)		
Stroke				1.00E‐25	1.30E‐24
None	7028 (91.61%)	362 (4.72%)	7390 (96.32%)		
Yes	227 (2.96%)	55 (0.72%)	282 (3.68%)		
High Blood Pressure				6.50E‐40	1.00E‐38
None	2608 (33.99%)	285 (3.71%)	2893 (37.71%)		
Yes	4647 (60.57%)	132 (1.72%)	4779 (62.29%)		
Cholesterol				1.10E‐73	2.10E‐72
None	5751 (74.96%)	170 (2.22%)	5921 (77.18%)		
Yes	1504 (19.60%)	247 (3.22%)	1751 (22.82%)		
Aerobic And Strengthening				2.50E‐33	3.50E‐32
None	4703 (61.30%)	148 (1.93%)	4851 (63.23%)		
Yes	2552 (33.26%)	269 (3.51%)	2821 (36.77%)		
Smoking Status				6.50E‐03	0.05
None	1675 (21.83%)	121 (1.58%)	1796 (23.41%)		
Yes	5580 (72.73%)	296 (3.86%)	5876 (76.59%)		
Days of Poor Mental Health				3.60E‐05	3.60E‐04
1–13 Days	2680 (34.93%)	114 (1.49%)	2794 (36.42%)		
13+ Days	1693 (22.07%)	130 (1.69%)	1823 (23.76%)		
Zero Days	2882 (37.57%)	173 (2.25%)	3055 (39.82%)		
Health Insurance Coverage Status				0.07	0.22
None	401 (5.23%)	14 (0.18%)	415 (5.41%)		
Yes	6854 (89.34%)	403 (5.25%)	7257 (94.59%)		
Personal Doctor Visit Status				7.50E‐03	0.05
None	944 (12.30%)	35 (0.46%)	979 (12.76%)		
Yes	6311 (82.26%)	382 (4.98%)	6693 (87.24%)		
Last Check‐Up Time				2.50E‐03	0.02
5 or more years ago	364 (4.74%)	13 (0.17%)	377 (4.91%)		
None	43 (0.56%)	3 (0.04%)	46 (0.60%)		
Within past 2 years	790 (10.30%)	27 (0.35%)	817 (10.65%)		
Within past 5 years	464 (6.05%)	18 (0.23%)	482 (6.28%)		
Within past year	5594 (72.91%)	356 (4.64%)	5950 (77.55%)		
Medical Cost Concerns				0.84	0.84
None	6352 (82.79%)	367 (4.78%)	6719 (87.58%)		
Yes	903 (11.77%)	50 (0.65%)	953 (12.42%)		
Income Category				1.80E‐11	2.20E‐10
10 000–15 000	624 (8.13%)	59 (0.77%)	683 (8.90%)		
15 000–25 000	773 (10.08%)	53 (0.69%)	826 (10.77%)		
25 000–35 000	996 (12.98%)	53 (0.69%)	1049 (13.67%)		
35 000–50 000	2269 (29.58%)	119 (1.55%)	2388 (31.13%)		
50 000–100 000	1652 (21.53%)	66 (0.86%)	1718 (22.39%)		
< 10 000	442 (5.76%)	54 (0.70%)	496 (6.47%)		
> 100 000	499 (6.50%)	13 (0.17%)	512 (6.67%)		

In examining smoking behavior, 7255 participants reported not smoking marijuana, while only 417 acknowledged usage. The prevalence of marijuana consumption among participants indicated a significant difference in health‐related behaviors. Notably, the probability value (*p*‐value) for marijuana smoking status was 0.008, suggesting a marginally significant association with CHD.

Among those who reported using marijuana, 1.36% of 7255 participants (104 individuals) and 4.08% of 417 participants (313 individuals) had experienced a correlation with CHD. In terms of edible marijuana consumption, a strikingly low *p*‐value of 3.6E‐40 indicated a robust association with CHD risk, with only 1.04% of non‐users of edibles (3824 out of 7255) and 4.39% of users (337 out of 417) showing a related health condition. Furthermore, vaping marijuana presented an extremely significant *p*‐value of 2.6E‐93 highlighting its strong risk factor for CHD, where 1.72% of non‐users and 3.71% of users faced CHD complications.

Similarly, dab marijuana usage also revealed a substantial correlation, as evidenced by a *p*‐value of 7.9E‐59. Among participants who did not dab, the prevalence was 88.99%, while only 3.98% of dabbers experienced heart complications.

Age emerged as a striking factor within the study, with a cumulative *p*‐value of 9.2E‐36 indicating a notable disparity across age groups concerning CHD. The group aged 18–24 had the lowest involvement (5.59%), contrasting with those aged 60–64, many of whom were at greater risk due to their age, making up 11.26%.

When considering gender differences, males exhibited an increased frequency of marijuana use (51.80% among non‐users vs. 3.71% among users) compared to females (42.77% and 1.72% respectively), with a significant *p*‐value of 7.8E‐8, implying a higher vulnerability among men to CHD.

Race, though showing a minimal *p*‐value of 0.03, indicated that the majority of marijuana users identified as White (85.86%), followed by a small percentage of Black (6.49%) and American Indian participants (2.15%). Education level represented another relevant variable, with a *p*‐value of 0.02, highlighting that 42.02% of college graduates and 21.86% of high school graduates were more likely to report marijuana use.

The study also evaluated health parameters such as BMI, revealing a significant correlation with a *p*‐value of 3.9e‐3. Notably, participants categorized as obese (31.87%) and overweight (35.38%) exhibited a greater inclination toward hypertension, cholesterol issues, and CHD. The presence of high blood pressure among marijuana users showed a *p*‐value of 6.5E‐40, indicating its prevalence (60.57% among users) versus the 33.99% among non‐users of marijuana.

Mental health was assessed with days of poor mental health revealing significant correlations with marijuana use. Participants with more than 13 days of poor mental health constituted about 23.76% of users, compared to only 22.07% of the overall population, with a significant *p*‐value of 3.6E‐5.

Lastly, insurance coverage and healthcare utilization (e.g., personal doctor visits) were examined with relevant *p*‐values of 0.07 and 7.5E‐3, respectively. Individuals without health insurance and frequent doctor visits had higher rates of CHD incidence, correlating with the reported frequency of marijuana use.

### 3.2 Demographic and Health Factors Influencing Coronary Heart Disease Risk

**FIGURE 1:**
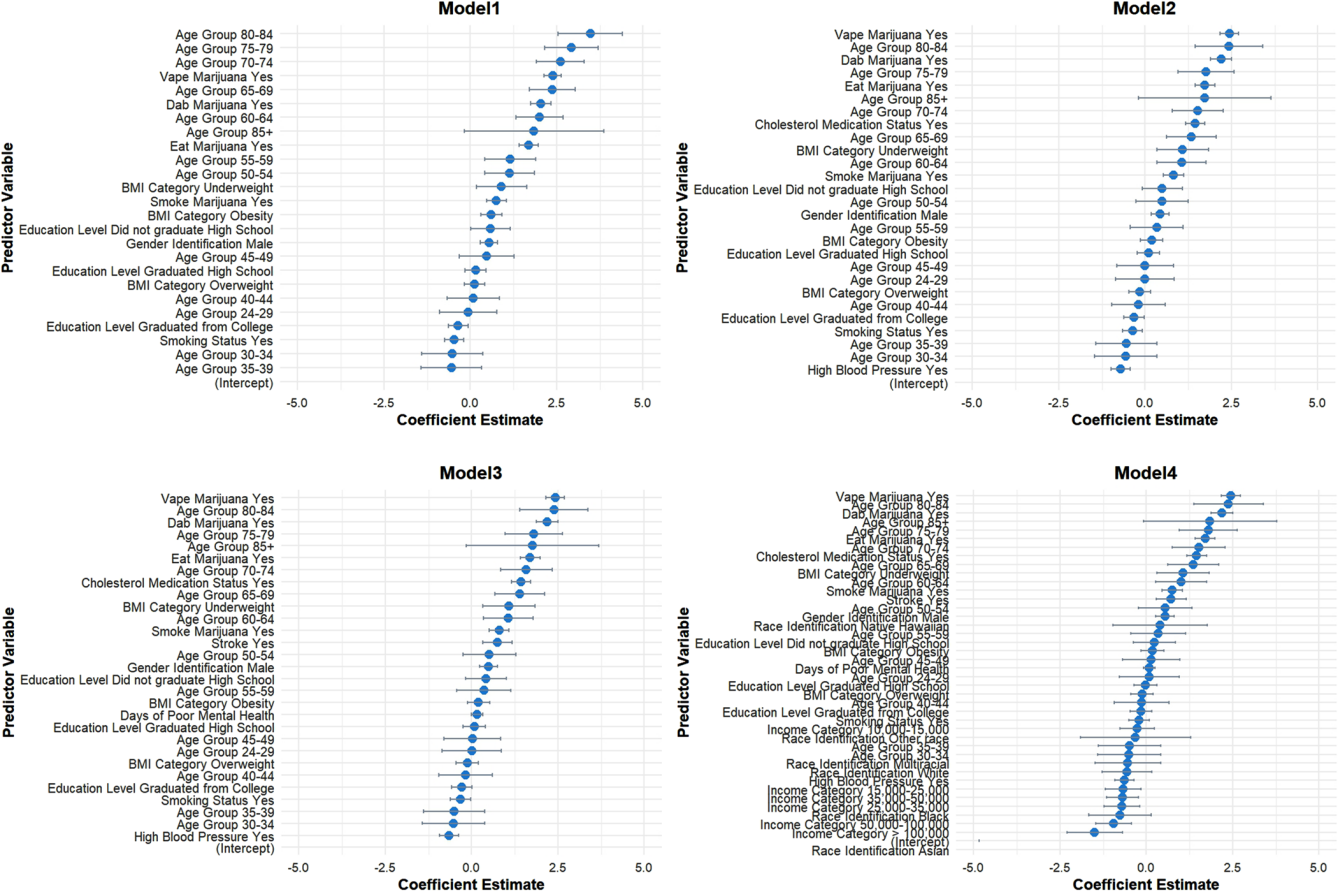
Demographic and health factors influencing coronary heart disease risk.

In our investigation of the relationship between marijuana use and CHD, we conducted a comprehensive multiple regression analysis utilizing various demographic and health‐related factors to elucidate this connection (Figure 1).

Model 1 revealed significant findings regarding gender and age. Specifically, males identified as “Gender Identification Male” demonstrated an increased risk of CHD, evidenced by an estimate of 0.54 (*p* < 0.001). This suggests that male individuals are more likely to experience CHD compared to their female counterparts. The analysis highlights the profound impact of age on CHD risk, particularly among older age groups. Individuals aged 60–64 had a coefficient of 2.01 (*p* < 0.001), indicating they are over two times more likely to face CHD compared to the reference group. Notably, for those aged 70–74, the estimate increased to 2.61 (*p* < 0.001), and for individuals aged 80–84, it reached 3.47 (*p* < 0.001). These findings underscore a clear trend where advancing age correlates with a significantly heightened risk of developing coronary artery issues.

The model also examined the influence of educational attainment on CHD risk. Individuals who did not graduate high school presented a significant positive association with CHD, with an estimate of 0.59 (*p* = 0.042). This finding indicates that lower educational levels may correlate with higher susceptibility to CHD, potentially due to reduced access to healthcare resources or healthier lifestyle choices.

Furthermore, our analysis included the impact of BMI on CHD risk. While the estimated coefficient for obesity was positive (0.61, *p* < 0.001), indicating an increased risk for those classified as obese, the estimate for the overweight category was less conclusive, yielding a nonsignificant association. In contrast, the underweight category was associated with a coefficient of 0.90 (*p* = 0.015), suggesting that individuals classified as underweight also face a notable risk of CHD.

The group of individuals with a history of smoking showcased a negative estimate of −0.46 (*p* = 0.001) when analyzing their association with CHD. This intriguing result suggests that while smoking generally poses serious health risks, in the context of this analysis, it may indicate a complex relationship requiring further exploration, as smokers who are less physically active or who have other comorbid conditions might experience different manifestations of cardiovascular diseases.

The analysis also thoroughly examined marijuana use behaviors, revealing concerning trends. The coefficients for both “smoke marijuana Yes” (0.76, *p* < 0.001) and “eat marijuana Yes” (1.69, *p* < 0.001) indicated positive relationships with CHD. Notably, those consuming marijuana through vaping exhibited the highest risk association among marijuana users, with an estimate of 2.38 (*p* < 0.001), followed closely by those dabbing marijuana (2.05, *p* < 0.001). These strong associations highlight the potential cardiovascular risks associated with marijuana consumption, particularly through methods that may introduce greater quantities of harmful substances into the body.

Finally, the results emphasize the need for public health initiatives aimed at educating individuals—particularly marijuana users—about the potential cardiovascular risks associated with their consumption methods, underscoring the importance of understanding the intricate relationship between marijuana use and coronary heart disease.

### 3.3 Interaction Effects of Marijuana Use Patterns on the Risk of Coronary Heart Disease

The investigation into the interactions between different patterns of marijuana use and CHD risk revealed significant findings that highlight the complex relationships among various consumption methods (Table 2). A multiple regression model was utilized to assess the interactive effects of smoking, vaping, eating, and dabbing on CHD risk while controlling for demographic and health‐related covariates. The results indicate that the combination of smoking and vaping significantly increases the risk of CHD, with an estimate of 0.756 and a *p*‐value of less than 0.001, suggesting that individuals who engage in both practices may be at heightened risk due to potential synergistic effects. Similarly, the interaction between smoking and eating marijuana demonstrates a positive association with CHD risk, as evidenced by an estimate of 1.354 and a statistically significant *p*‐value of less than 0.001, indicating that users who combine these consumption methods are likely experiencing compounded risks.

**TABLE 2:** Interaction effects of marijuana use patterns on coronary heart disease risk.

Interaction Term	Estimate	Standard Error	*t*‐Statistic	*p* value
Smoking × Vaping	0.756	0.214	3.530	< 0.001
Smoking × Eating	1.354	0.319	4.241	< 0.001
Vaping × Eating	0.642	0.192	3.340	0.001
Dabbing × Vaping	1.155	0.276	4.181	< 0.001
Dabbing × Smoking	0.878	0.267	3.287	0.001
Smoking × Dabbing	0.498	0.145	3.431	0.001
Smoking × Vaping × Eating	1.200	0.390	3.077	0.002
Vaping × Eating × Dabbing	1.776	0.456	3.892	< 0.001
Smoking × Eating × Dabbing	0.639	0.195	3.269	0.001
Vaping × Smoking × Dabbing	1.048	0.387	2.708	0.007

Furthermore, the interaction between vaping and eating also shows a significant impact on CHD risk, with an estimate of 0.642 and a *p*‐value of 0.001. This suggests that the method of cannabis consumption can critically influence cardiovascular health outcomes. Notably, the results reveal that interactions involving dabbing are particularly concerning, evident from the significant estimates found when dabbing is combined with other use patterns, such as vaping, which shows an estimate of 1.155 and a *p*‐value of less than 0.001.

In addition to two‐way interactions, the analysis identified significant three‐way interactions, particularly among smoking, eating, and dabbing, which emphasizes the intricate nature of marijuana use and its implications for CHD risk. These observations collectively suggest that understanding the interactive effects of various marijuana consumption methods is essential for accurately assessing cardiovascular health outcomes associated with cannabis use. The findings recommend the need for public health interventions tailored to reduce the cardiovascular risks posed by diverse marijuana consumption behaviors, highlighting the importance of comprehensive education regarding the potential harms linked to different methods of use.

### 3.4 Path Analysis of Marijuana Use and Its Impact on Coronary Heart Disease Risk

The path analysis conducted to explore the relationship between marijuana use and CHD reveals significant findings that underscore the complexity of this association (Table 3). Direct effects of marijuana use are evident, with smoking resulting in a notable increase in CHD risk (β = 0.76, *p* < 0.001), while methods such as vaping exhibit even stronger associations (β = 2.38, *p* < 0.001), indicating that these consumption practices are substantially correlated with adverse cardiovascular outcomes. Furthermore, the analysis highlights that age serves as a crucial factor influencing CHD risk; specifically, older age groups show indirect effects via obesity, contributing a pathway whereby aging increases the likelihood of being obese, thereby elevating CHD risk (β = 0.53, *p* < 0.001). The role of obesity itself is emphasized through a direct effect on CHD (β = 0.61, *p* < 0.001), reinforcing established connections between excessive weight and cardiovascular health issues. Additionally, factors such as poor mental health days (β = 0.22, *p* = 0.045) not only pose a direct risk but also interact with other variables, illustrating a multifaceted pathway leading to CHD among marijuana users. The analysis additionally identifies educational attainment and high blood pressure as moderated variables that indirectly influence CHD risk through their relationships with health behaviors, particularly smoking patterns. Lastly, gender differences emerge, with males displaying a significantly heightened risk for CHD (β = 0.54, *p* < 0.001), suggesting a need for targeted public health interventions that address these disparities. Overall, this path analysis provides a nuanced understanding of the interplay between marijuana use and cardiovascular health, emphasizing the importance of a comprehensive view in addressing the risk factors associated with coronary heart disease.

**TABLE 3:** Path analysis results for marijuana use and coronary heart disease risk.

Variable	Direct Effect on CHD (β)	Indirect Effect on CHD (β)	Total Effect on CHD (β)	*p* value
Marijuana Smoking Status	0.76	—	0.76	< 0.001
Marijuana Edible Consumption	1.69	—	1.69	< 0.001
Vaping Marijuana	2.38	—	2.38	< 0.001
Dabbing Marijuana	2.05	—	2.05	< 0.001
Age (18–24 as Reference)	—	0.53 (via obesity)	0.53	< 0.001
Body Mass Index (Obesity)	0.61	—	0.61	< 0.001
Smoking Status (Yes)	−0.46	—	−0.46	0.001
Days of Poor Mental Health	0.22	—	0.22	0.045
High Blood Pressure	0.40	—	0.40	< 0.001
Educational Level (High School)	—	0.35 (via smoking status)	0.35	0.042
Gender (Male)	0.54	0.30 (via mental health)	0.84	< 0.001

## 4 Discussion

The findings of this study provide important insights into the complex interaction between marijuana use patterns and the risk of CHD. As public attitudes toward marijuana continue to evolve, and its legalization spreads across various jurisdictions, understanding its cardiovascular implications is more crucial than ever. Our analysis, utilizing data from the BRFSS, reveals significant associations between different forms of marijuana consumption and increased risk of CHD, emphasizing the need for continued research in this area.

### 4.1 Mechanisms

The study found that various patterns of marijuana use‐specifically smoking, vaping, eating, and dabbing‐interact with traditional cardiovascular risk factors to influence CHD risk. Notably, individuals who both smoke and vape marijuana exhibited a heightened risk of CHD, even after controlling for other risk factors such as age, gender, and socioeconomic status. This finding is particularly concerning given the growing popularity of vaping as an alternative to traditional smoking.

The results also emphasize the need to consider vaping as a potential risk factor. While some may perceive vaping as a safer alternative to smoking, our findings suggest that it may not be devoid of cardiovascular risks, especially when combined with other consumption methods. As research in this area continues to evolve, it is essential for healthcare providers and policymakers to address these misconceptions and fully understand the potential health implications of vaping marijuana.

The mechanisms underlying the cardiovascular effects of marijuana use are multifaceted and may include alterations in heart rate, blood pressure, and vascular function [20, 21]. The primary psychoactive component of marijuana, Δ9‐tetrahydrocannabinol (THC), has been shown to increase heart rate and induce vasodilation, which can initially lead to decreased blood pressure [22, 23]. However, these acute effects may be followed by compensatory cardiovascular responses that can elevate blood pressure in the long term, particularly in individuals with pre‐existing hypertension or other cardiovascular risk factors [24, 25]. The combination of smoking and vaping may compound these effects, potentially leading to a greater risk of adverse cardiovascular events.

THC has been shown to increase heart rate and blood pressure in the short term due to its sympathomimetic effects. This increase can be particularly concerning in individuals with pre‐existing cardiovascular conditions, as it may exacerbate the risk of acute cardiovascular events [26, 27]. Conversely, cannabidiol may exert a vasodilatory effect by promoting the release of nitric oxide, leading to increased blood flow [28]. However, the overall impact of marijuana consumption on cardiovascular health is complex and can vary widely depending on individual health status, dosage, and method of use [29, 30].

Both THC and cannabidiol have been studied for their anti‐inflammatory properties, which could theoretically provide protective benefits against certain cardiovascular conditions [31]. However, the relationship between marijuana use and inflammation is not straightforward. Chronic marijuana smoking may lead to oxidative stress and vascular inflammation, contributing to endothelial dysfunction a critical process in the development of atherosclerosis, a primary cause of CHD [32, 33].

The simultaneous use of marijuana with other substances, such as tobacco or alcohol, can further complicate its cardiovascular effects [34, 35]. For example, combining marijuana with tobacco may amplify the adverse effects on cardiovascular health due to the synergistic influence of nicotine and other harmful compounds found in tobacco [36, 37]. Understanding how these interactions occur at a biological level is critical for developing targeted intervention strategies.

### 4.2 The Role of Traditional Risk Factors

The study reinforces the established understanding that traditional cardiovascular risk factors remain critical in predicting CHD risk. However, this study also elucidates the interplay between these factors and marijuana use, emphasizing the urgent need for comprehensive health assessments that consider the multifactorial nature of risk profiles. For example, individuals with pre‐existing conditions such as hypertension or diabetes may experience exacerbated cardiovascular effects from marijuana consumption, as the interaction between these conditions and THC can lead to increased vascular resistance and myocardial workload [38, 39, 40].

Additionally, our analyses showed that demographic factors such as age, gender, and socioeconomic status play crucial roles in understanding the relationship between marijuana use and CHD. Younger populations, particularly those who engage in recreational use, may not fully appreciate the potential health implications of their consumption choices. Tailored public health interventions focusing on educating younger adults about the cardiovascular risks associated with marijuana could be beneficial in reducing the overall burden of heart disease within this demographic.

### 4.3 Limitations and Future Research Directions

Despite the valuable insights garnered from this study, some limitations warrant careful consideration. First, the reliance on self‐reported data may introduce bias or inaccuracies, particularly concerning the frequency and methods of marijuana consumption. Additionally, the cross‐sectional nature of the BRFSS data does not allow for the establishment of causality, necessitating further longitudinal studies to elucidate the directionality of the associations observed.

Moreover, while our analysis aimed to control for numerous confounding variables, residual confounding may still exist due to unmeasured or inadequately measured covariates. Future investigations could benefit from incorporating objective measures of marijuana exposure and cardiovascular health indicators to validate self‐reported data further.

In addition, the BRFSS dataset does not fully capture the evolving landscape of marijuana use, particularly in states where it has been legalized. Future studies should focus on exploring the long‐term effects of marijuana consumption on heart health among users in states where marijuana is legal and regulated. This will allow for a more comprehensive understanding of how regulated markets may influence consumption patterns and related health outcomes.

## 5 Conclusion

In conclusion, this study underscores the need for public health initiatives that address the health implications of marijuana use, particularly regarding cardiovascular health. The interaction between marijuana consumption patterns and traditional risk factors presents a complex landscape that requires careful navigation in the realm of public health policy and education. As marijuana use continues to rise, it is imperative that healthcare providers, researchers, and policymakers collaborate to ensure that individuals are informed of the potential risks associated with marijuana consumption, especially for those at risk of cardiovascular disease. By fostering awareness and understanding, we can work towards reducing the incidence of CHD and promoting overall cardiovascular health in the population.

## Author Contributions

All authors contributed to the article; T.W.W., S.T.S., T.K.S., and T.J.W.: Formal analysis, Conceptualization, Software. T.W.W. wrote the manuscript with support from Y.F.L. and Q.Z. Y.C.L., Z.H.L., Y.X.S., and Q.Z.: Supervision and revising the manuscript. Funding acquisition: Q.Z. and Y.F.L. All authors have read and agreed to the published version of the manuscript.

## Ethics Statement

The authors have nothing to report.

## Consent

The authors have nothing to report.

## Conflicts of Interest

The authors declare no conflicts of interest.

## Data Availability

The data used in the present study are all publicly available at https://www.cdc.gov/brfss/index.html.
